# New Hair Growth Cream Formulation with Cocoa Pod Peel (*Theobroma cacao* L.)

**DOI:** 10.1155/2022/2299725

**Published:** 2022-03-14

**Authors:** Resmi Mustarichie, Aliya Nur Hasanah, Gofarana Wilar, Dolih Gozali, Nyi Mekar Saptarini

**Affiliations:** ^1^Pharmaceutical Analysis and Medicinal Chemistry Department, Faculty of Pharmacy, Universitas Padjadjaran, Sumedang 45363, Indonesia; ^2^Pharmacology and Clinical Pharmacy Department, Faculty of Pharmacy, Universitas Padjadjaran, Sumedang 45363, Indonesia; ^3^Pharmaceutical and Technological Pharmacy Department, Faculty of Pharmacy, Universitas Padjadjaran, Sumedang 45363, Indonesia

## Abstract

Our previous study verified that the waste skin of cocoa (*Theobroma cacao* L) fruit or waste cocoa pod husks had the efficacy to overcome hair loss or alopecia. This study aims to determine the formula and activity of hair cream of cocoa pod peel water fraction, which is effective in stimulating hair growth. Activity testing uses the modified Tanaka method. The results showed that the cocoa husk wastewater fraction could be formulated into hair cream, but there were changes in viscosity and pH after the freeze-thaw test, but still within the allowed limit. The hair cream water fraction gel stimulated hair growth activity based on the hair length data with a significant difference in concentration of the preparation. The best activity in hair cream preparation was at 12.5% concentration. In addition, there were no signs of irritation to the rabbit's skin where hair cream preparation was applied. The results of this study indicated that cocoa fruit peel cream can be used for antialopecia treatments.

## 1. Introduction

Hair is one of the body's vital parts because it is an essential factor in appearance and confidence. The role of hair is quite important both in humans and animals due to its functions to protect from sunlight, UV rays, regulate temperature, and sweat evaporation. Alopecia is a dermatological disorder that has been recognized for more than 2000 years. Alopecia is baldness, which involves absence or hair loss, especially the part of the head [1–3]. Research on synthetic treatments is still in development, but it should be noted that the side effects of synthetic drugs cannot be ignored. Thus, herbal medicine is a possible alternative to synthetic drugs. Natural medicine has been used for generations in several centuries to treat alopecia [4]. Traditional medicine is also a treatment that most Indonesian choose. Traditional medicine is quite popular, based on the study results, not causing too many side effects [5].

Indonesia is the third largest cocoa (*Theobroma cacao* L.) producing country globally after Ivory Coast and Ghana, with production that continues to grow every year [6, 7]. Therefore far, cocoa waste has not been used except for fertilizer and fodder. The content of the skin (husk) is rich in phenolic compounds [8]. The people of the Dingga Linggarjati, who live at the foot of Mount Galunggung, West Java, Indonesia, use the cocoa pod husks' skin to overcome baldness and several other skin problems. The skin of cocoa fruit is usually boiled, and then, the water is clamped and taped to treat the head of children who are corroded and bald. The absence of scientific research on the properties of the cocoa skin is the rationale for this research. Our previous study verified that the waste skin of cocoa (*Theobroma cacao* L) fruit or waste cocoa pod husks had the efficacy to overcome hair loss or alopecia [9]. To find the potential antialopecia constituents from *Theobroma cacao*, an in silico study verification has been carried out [10, 11]. This work reports on the new hair cream formula of cocoa pod peels which effectively stimulate hair growth.

## 2. Materials and Methods

The research methods include collecting cocoa pods, processing, cocoa pods, extraction using 70% ethanol solvent by maceration, fractionation of cocoa pod extract using the ECC (liquid-liquid extraction) method, formulation of cream preparations of cocoa rind water fraction, evaluation of cream preparation formula, and cream activity test for male rabbits based on the Tanaka method (1980) [12], irritation test, and data processing.

### 2.1. Plant Material Processing

Cocoa pods were separated and washed thoroughly, drained to dry, cut into smaller parts, dried simply by cooling air, and protected from direct sunlight.

### 2.2. Extraction

Five hundred grams of cocoa pods were put into a macerator; then, 1250 mL of 70% ethanol solvent was added and tightly closed and protected from direct sunlight. Extraction was carried out for 3 × 24 hours. Every 24 hours, the macerate was collected, and the solvent was changed. The macerate was then concentrated using a rotary evaporator 40°C until a thick ethanolic extract was obtained [13].

### 2.3. Fractionation

The method used for fractionation was a liquid-liquid extraction method using n-hexane and ethyl acetate as solvents. The water fraction obtained was then used for the formulation of cream preparations [13].

### 2.4. Cocoa Pod Skin Water Fraction Cream Formulation

Three formulas were developed with various extract concentrations.

### 2.5. Evaluation of Cream Preparations

The following are the testing parameters for the cocoa peel extract cream preparation:

1. Organoleptic test: the shape of the cream, the color, and smell of the cream was observed [14].
2. Spreadability test: according to the method by Garg et al. [15].
3. Homogeneity test: conducted based on Cooper et al. [16].
4. pH test: the pH measurement was carried out to determine the suitability between the pH of the preparation and pH of the skin. Cream pH testing was carried out at room temperature. The cream preparation to be tested was diluted first using distilled water in a ratio of 1 : 10. The pH meter was calibrated using a buffer solution of pH seven and pH 4 [14].
5. Cream type test: to see whether the type is water-in-oil (W/O) or oil-in-water (O/W) [17].
6. Stability test: the stability test used the freeze-thaw method where the sample underwent a freeze and thaw cycle for 10 days with 5 cycles. Each cycle consisted of storage at 4°C and 40°C, for 24 hours, respectively [18].
7. Viscosity test: the viscosity test of the cream preparation was determined using a Brookfield viscometer [19].

### 2.6. Testing the Activity of Hair Growth Test Preparations on Test Animals

The following was a test of the activity of the cream of water fraction of cocoa pods as a hair growth preparation using the Tanaka method (1980) with modifications [20]:

#### 2.6.1. Animal Preparation

The animals tested in this study were male rabbits aged 4-5 months with a bodyweight of 2–2.5 kg. The number of rabbits used is obtained from the calculation of the Federer formula, namely, (*t* − 1) (*n* − 1) 15, where *t* is the number of treatments, and *n* is the number of repetitions of each treatment.

Rabbits were acclimatized for 7 days to live in new environmental conditions and new treatments. The back of the rabbit was cleaned of hair and then smeared with 70% alcohol. The rabbits were then rested for up to 24 hours before treatment [9].

### 2.7. Rabbit Grouping

The back of the rabbit was divided into several plots as follows:

- Box 1: normal control (no treatment)
- Box 2: positive control (minoxidil 5.0%)
- Box 3: base control (cream base)
- Box 4: cream preparation of cocoa water fraction (7.5%)
- Box 5: cream preparation of cocoa water fraction (10.0%)
- Box 6: preparation of cocoa water fraction cream (12.5%)

Determination of the smearing area on the rabbit's back was carried out randomly, and it was hoped that hair growth activity in all regions with different representatives could be represented [21].

### 2.8. Treatment of Rabbits

The back of the rabbit was shaved clean of hair and divided into several plots, which would later be smeared with test and control preparations. The plot was a box with a size of 2 × 2 cm marked with a line; the distance of each box was 1 cm. The dosage (0.5 g each) was administered twice daily, in the morning and evening. The five longest strands of rabbit hair were measured every three days in each box, namely, on days 3, 6, 9, 12, 15, and 18. The length of the rabbit hair was measured using a caliper [9].

### 2.9. Irritation Test

An irritation test was done by observing changes that occurred on the skin of rabbits where creams were applied. The skin was smeared with the preparation twice a day until day 18. The irritation test by monitoring changes that occur on the skin was carried out on days 0, 3, 6, 9, 12, 15, and 18.

### 2.10. Data Processing

The results of measuring the length of the rabbit's hair obtained were then averaged. The normality test was performed using the Shapiro–Wilk method and homogeneity using Levene's approach, and then, the data were processed using the Kruskal–Wallis statistical method.

## 3. Results

The results of the study are provided in Tables 1–4.

## 4. Discussion

### 4.1. Simplicia Extraction by the Maceration Method

The extraction process aimed to extract secondary metabolites from simplicia of cocoa pods. The solvent used was 70% ethanol because it is nontoxic, cheap, easy to obtain, and sufficient water content allows the solvent to easily penetrate simplicia, so that ethanol can easily extract the metabolites in simplicia. Simplicia as much as 1250 g in a macerator soaked with 500 ml of 70% ethanol was left overnight. Every 24 hours, simplicia was stirred and replaced with new solvent up to three times. The liquid extract was then concentrated using an evaporator at 40°C to evaporate ethanol. The extract was then stored in a waterbath until a thick extract was formed. After that, the extract yield was calculated, and the yield was 12.6% [9].

### 4.2. Fractionation by the Liquid-Liquid Extraction Method

Fractionation was a method of separating compounds according to their polarity. The method used for LLE cocoa pod skin fractionation was based on our previous publication. This method has the principle of like dissolves, and a compound will be attracted to a phase with the same level of polarity as the compound. In this method, three solvents were used, namely, distilled water, ethyl acetate, and n-hexane. Aqueous as a polar phase, ethyl acetate was a semipolar phase, and n-hexane was a nonpolar solvent. Components that dissolve in the nonpolar phase will dissolve in n-hexane, while the polar phase will dissolve in distilled water. The water fraction had the most concentrated color, while the ethyl acetate fraction was yellow and the n-hexane fraction was pale yellow. The water fractions of the cocoa pods were then concentrated using an evaporator and a waterbath, so that the solvent could evaporate to form a thick extract. The thick extract of the cocoa pod skin was dark brown with a characteristic odor [9].

### 4.3. Results of the Cream Formulation

The following was the formula and procedure for making water fraction cream of cocoa pods (Table 1).

Formulas were arranged based on the formulation [17].

Stearic acid, cetyl alcohol, triethanolamine, liquid paraffin, glycerin, methylparaben, propylparaben, and distilled water were weighed. The oil phase consisting of stearic acid, cetyl alcohol, and liquid paraffin was heated at 70°C until all parts melted. The aqueous phase, namely, glycerin, triethanolamine, methylparaben and propylparaben, and distilled water, was heated in a glass beaker on a hot plate at 70°C until melted. The water phase was slowly added to the oil phase while constantly stirring with a 500 rpm mechanical stirrer until a homogeneous cream phase was formed. After the base was formed, the water fraction of the cocoa peel was added to the cream base, and the preparation was stirred again until homogeneous. In the making of cream preparations, the aqueous phase and the oil phase ought to be at the same temperature conditions, so that the cream base could be appropriately formed, and there was no phase separation. In the manufacturing process, the cream was directly formed during the process of mixing the water and oil phases with constant stirring at 70°C. The cream base was white, and the color changed to light brown after the addition of the water fraction of the cocoa pods. The result of the cream of water fraction of cocoa pods had a white and thick consistency.

### 4.4. Excipient Profile in Cream Formulation [22]

Stearic acid is an emulsifier as an oil phase base and is helpful for increasing emulsion stability. Steric acid is nontoxic and does not irritate the skin. In cream preparations, the concentration of stearic acid commonly used is between 1 and 20%. Stearic acid is frequently used with triethanolamine (TEA). TEA is used as an anionic emulsifier and serves to increase the pH of the preparation. TEA has hygroscopic properties. Liquid paraffin is used as an excipient for pharmaceutical preparations. This substance functions as an emollient and is commonly used in oil-in-water emulsion types. Cetyl alcohol is an odorless and tasteless substance in the form of white flakes. Cetyl alcohol 1–5% concentration serves as an emollient in creams. It can improve the stability and consistency of the preparation and affect softening of the skin. Glycerin is a colorless and odorless viscous solution—glycerin functions as a humectant and emollient. In cream preparations, glycerin can also function as a solvent and cosolvent. The concentration of glycerin used as a humectant and emollient is 30%.

Nipagin and Nipasol are used as preservatives or antimicrobial substances in cosmetics, food, and pharmaceutical preparations. Nipagin and Nipasol are effective as antimicrobials at pH 4–8.

### 4.5. Cream Preparation Evaluation Results

The following are the results of the evaluation of the preparation of cocoa pod peel extract cream.

### 4.6. Organoleptic Test

The organoleptic test aims to see the physical appearance of the cream. The water fraction cream of the cocoa pods at 7.5%, 10%, and 12.5% concentrations showed a thick and light brown color. The higher the concentration of the fraction, the more concentrated the color. The cream had a characteristic odor of the cocoa pod water fraction, but with the addition of lavender perfume, the characteristic odor of the fraction was masked. The creamy formula did not experience any change in smell or rancidity. A rancid odor in cream can occur due to oxidizing fats or oils, and light can accelerate the oxidation reaction. The cream formula used did not contain fatty substances that had double bonds, so that the tested formula did not cause a rancid odor. Other researchers [23] also applied the organoleptic test.

### 4.7. Homogeneity Test

This was done by applying 0.1 g of cream evenly and thinly on the watch glass. The results of the preparation of nail water root fraction cream were 7.5%, 10%, and 12.5% and showed homogeneous results as evidenced by the evenly distributed cream color and the absence of spots. This test was also carried out by other researchers [24].

### 4.8. pH Test

The pH measurement was carried out to determine the suitability between the pH of the preparation and pH of the skin. A suitable pH ranges 4.5–6.5. Cream pH testing was carried out at room temperature. The cream preparation was diluted first using distilled water in a ratio of 1 : 10. The pH meter was calibrated using a buffer solution of pH 7 and pH 4. The electrodes on the pH meter are then dipped into the sample solution to be examined, and the pH results on the screen were recorded [14]. The pH of the cream preparations of nail root water fraction with concentrations of 7.5%, 10%, and 12.5% was 6.27, 6.1, and 5.85. This indicates that the preparation has a pH value within the requirements. A pH of 7 is chemically neutral. Haircare products with a pH value between 3.5 and 5.5 are considered beneficial for haircare products.

When the pH value drops below 6.0, the cuticle layer contracts and tightens; a mild acidic conditioner (lower than pH 7) can help to add shine to the hair, as a smooth surface can reflect more light; strong acids will however damage the hair. Acid conditioners should not be used when red/orange tones have been added to the hair. Instead, we use a color-specific care range.

When the pH value becomes more alkaline (above 7.0), the cuticle layer softens and expands like a pinecone [25].

### 4.9. Spreadability Test

The spreadability test was used to determine the spreadability of the cream preparation when applied to the skin [26]. The results of the dispersion test on the cream preparation of the water fraction of cocoa pods are given in Table 2.

### 4.10. Cream Type Test

The cream preparation was placed into a cup, and then, a drop of oil-soluble dye solution was added and mixed. If there was a homogeneous red color in the outer phase of the cream, then the type of cream was water in oil (w/o). The test results showed that there was an inhomogeneous distribution when the cream preparation was dripped with an oil-soluble dye solution. The cream preparation was then placed into a different cup. Drops of methylene blue were mixed evenly with the cream and then observed. The results showed that the preparation became a homogeneous blue color. The test results by diluting the cream using Aquadest indicated that the cream could be dissolved. Therefore, the type of cream was the water fraction of cocoa pods, namely, oil-in-water (o/w). The O/A cream type is a type of cream that was easy to wash off [27].

### 4.11. Viscosity Test

The viscosity test aims to determine the viscosity of the preparation. The results of the viscosity test of the cream with formula 1 (water fraction of cocoa pond 7.5%) were 105460 cps, formula 2 (with 10% fraction) 107876 cps, and formula 3 (with 12.5%) 114340 cps. The viscosity and surface tension of cream decreased with increasing temperature; therefore, the temperature affected the introduction of air bubbles [28].

### 4.12. Stability Test

The results showed no change in the cream in the organoleptic and homogeneity tests using the freeze-thaw method [18]. However, the pH value changed after the freeze-thaw stability test. The most significant change in the pH value occurred in cream preparations with 12.5% concentration of water fraction of cocoa pods, and more fractions could cause this in the preparation. However, all creams with varying concentrations had a pH that was still within the required range of 4.5–6.5. Viscosity changes also occurred in the cream after the freeze-thaw test; the viscosity of the cream decreased after the freeze-thaw stability test presumably caused by the water content in the cream, which was affected by extreme temperature changes, so that the cream viscosity decreased.

### 4.13. Hair Growth Activity Test

The activity test aimed to determine the hair growth stimulant activity of the cream of water fraction of cocoa pods. The activity test used the Tanaka method and used three male rabbits aged 4-5 months. Male rabbits were chosen because they are unaffected by the menstrual cycle or hormones that can affect hair growth. As well as, 4-5 months old rabbits weighing 1.5–2.5 kg were chosen because their physical properties enable testing and their back surface area allows for the application of groups of test preparations. Rabbits were first acclimatized for seven days to adapt to their environment and not to experience stress that could affect hair growth. After that, the rabbit's back was shaved, and alcohol was applied afterward to prevent irritation of the skin. After 24 hours, the rabbits were smeared with the test preparation on each test box.

**Figure 1:**
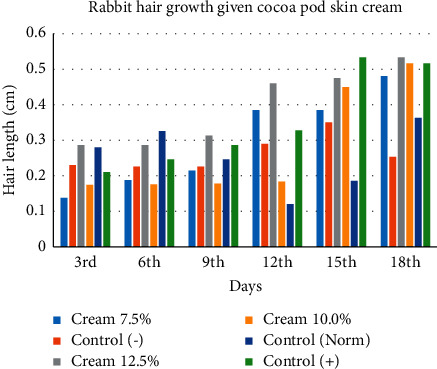
Rabbit hair length with cocoa pod skin cream.

The concentrations of water fractions of cocoa pods used were 7.5%, 10.0%, and 12.5%. The choice of concentration was based on the dose test according to previous studies [29, 30]. The results of testing hair growth stimulating activity in rabbits (Table 3 and Figure 1) showed that positive control and test preparations had better hair growth than normal control and negative control.

The SPSS test was used to determine the significance of rabbit hair length after the preparation was applied. The Shapiro–Wilk normality test was used to assess the normality of the rabbit hair length data; then, Levene's homogeneity test was carried out. The results showed that the rabbit hair length data are typically distributed as evidenced by the *P* value >0.05, and the homogeneity test shows the *P* value >0.05, which indicates the data are homogeneous. For this reason, further tests were carried out using the ANOVA method. The results showed that there was no significant difference in rabbit hair growth.

### 4.14. Irritation Test

The irritation test was carried out to determine the reaction caused by the preparation after it was applied to the skin. Irritation test criteria are used based on the classification given in Table 4. Based on the results of the study, there was no irritation reaction on the skin when the preparation was applied on the first day. However, after the preparation was used twice a day until the 6th day, there was a reddish color on the part of the skin that was given the preparation of cocoa pond water fraction cream 7.5%, 10%, and 12.5%. The reddish color occurred more quickly in the negative control preparation, namely, on the 3rd day. The slightly reddish color that appears on the rabbit's skin after application on the sixth day was thought to be due to the presence of excipients, namely, triethanolamine, Nipagin, and Nipasol, which were slightly irritating to the skin [31].

So far, there is no formulation of cocoa pod husk (*Theobroma cacao* L.) as a hair growth drug. The waste from the cocoa itself, which is the skin of the cocoa fruit, is not inferior to the cocoa beans, which are very commonly consumed by the community. From Vriesmann's research, polyphenols contained in cocoa pods are expressed as one of the content macromolecules such as carbohydrates, proteins, and fats, which is 4.6%, where they contain 1.32% greater than polyphenols contained in cocoa beans [32]. The study analyzed soluble polyphenols using spectrophotometry according to the Lecumberri method [33]. In other journals, the polyphenol content of cocoa pods was 5.78% [38]. It can also be noted that the possibility of different polyphenol contents can be caused by differences in the origin of the cocoa fruit used; in Vriesmann's research, the cocoa originates from Brazil, while the Ritaliah study originates from Indonesia. However, the data obtained state that the content of polyphenols in cocoa pods is one of the most prominent ingredients. More reports have provided support for nutritional and antioxidant therapies in the last few decades as a more effective and safer treatment option for hair loss. Various dietary supplements such as those enriched in plant extract, amino acids, and vitamins are popular free products. They are indeed proven to accelerate and prolong the anagen phase time in hair loss pattern (PHL) patients [34].

Based on our research, the cocoa fruit pod husk extract has activity as a hair growth stimulator in rabbits. Various research sources support this. The highest range in cocoa pods is polyphenols. The content of polyphenols in green tea can inhibit the activity of androgen receptors that cause hair loss. Furthermore, it can be estimated that the skin of cocoa fruit can potentially overcome the problem of hair loss, especially in androgenetic alopecia. We previously showed that compared to the positive control minoxidil, which is a drug to overcome hair loss, cocoa pod husks starting from 15% concentration of water extract significantly increased hair growth activity [9]. Therefore, from existing data, cocoa pod husks can be an alternative treatment for hair loss or alopecia. This should be considered because it cannot raise the value of the cocoa fruit itself. Not only do the seeds have millions of benefits, now the skin, which is a waste, can be developed as an alternative treatment for various diseases. Thus, from these data, it can be considered that the skin of cocoa fruit, which is rich in antioxidants and polyphenols, can help overcome hair damage, hair loss, and alopecia. Compared with tea and apple leaf plants, the skin of cocoa fruit has a higher polyphenol content. It has excellent potential as an alternative treatment for dermatologists, one of which is antialopecia. Therefore, this research suggests that cocoa pod skin cream with a concentration of 12.5% can be used for growth as an antialopecia.

## 5. Conclusion

Irrespective of the effects, hair growth proven by synthetic drugs is questionable due to the lack of efficacy, safety, or side effects of these drugs. Herbal medicine can provide a new revolution for hair growth today. Formulation of 12.5% cocoa pod skin cream has recently been found to stimulate hair growth in rabbits, which will help treat hair loss or alopecia [35].

## Figures and Tables

**Table 1:** Cream formulation of cocoa pods.

Ingredients	Concentration % (W/W)			
	Cream base	Formula 1	Formula 2	Formula 3
Cocoa pods fraction	—	7.5	10.0	12.5
Stearic acid	10.0	10.0	10.0	10.0
Cetyl alcohol	4.0	4.0	4.0	4.0
Triethanolamine	1.4	1.4	1.4	1.4
Liquid paraffin	5.0	5.0	5.0	5.0
Glycerine	5.0	5.0	5.0	5.0
Methylparaben	0.1	0.1	0.1	0.1
Propylparaben	0.05	0.05	0.05	0.05
Perfume	qs	qs	qs	qs
Distilled water	ad 100	ad 100	ad 100	ad 100

**Table 2:** Dispersion test on the formula.

Formula	Spreading
F1	3.86
F2	3.80
F3	3.76

**Table 3:** Cream activity on rabbit hair growth.

Treatment	Average length (cm) ± SD					
	Days					
	3	6	9	12	15	18
Control (−)	0.230 ± 0.162	0.226 ± 0.025	0.226 ± 0.037	0.29 ± 0.072	0.350 ± 0.113	0.253 ± 0.075
Control (norm)	0.138 ± 0.027	0.188 ± 0.005	0.215 ± 0.055	0.385 ± 0.123	0.530 ± 0.217	0.581 ± 0.016
Cream (fraction 7.5%)	0.138 ± 0	0.188 ± 0.005	0,215 ± 0.0208	0.385 ± 0.0404	0.385 ± 0.086	0.481 ± 0.132
Cream (fraction 10.0)	0.175 ± 0	0.178 ± 0.025	0.178 ± 0.0208	0.184 ± 0.017	0.45 ± 0.023	0.516 ± 0.047
Cream (fraction 12.5%)	0.286 ± 0	0.286 ± 0.025	0.313 ± 0	0.46 ± 0.065	0.475 ± 0.225	0.533 ± 0.028
Control (+)	0.210 ± 0.124	0.246 ± 0.071	0.286 ± 0	0.328 ± 0.276	0.453 ± 0.056	0.516 ± 0.061

**Table 4:** Stability test results.

Evaluation	Before freeze-thaw			After freeze-thaw		
	F1	F2	F3	F1	F2	F3
Organoleptic	Light brown, distinct smell	Light brown, distinct smell	Light brown, distinct smell	Light brown, distinct smell	Light brown, distinct smell	Light brown, distinct smell
Homogeneity	Homogenous	Homogenous	Homogenous	Homogenous	Homogenous	Homogenous
pH	6.27	6.1	5.85	6.10	5.93	5.51
Viscosity (cP)	105460	107876	114340	97933	107480	113020

## Data Availability

The data used to support this study are included within the article.

## Supplementary Materials

Supplementary Materials Supplementary description is material directly relevant to the conclusion of the study. This study consists of tables and figures, without which it is difficult to draw a discussion and conclusion from the article.
