# Supplementary material for: New Hair Growth Cream Formulation with Cocoa Pod Peel (Theobroma cacao L.)
Source: ScientificWorldJournal. 2022 Mar 14;2022:2299725. doi: 10.1155/2022/2299725 (PMC8938051; doi:10.1155/2022/2299725)
Supplement: Supplementary Materials — Supplementary description is material directly relevant to the conclusion of the study. This study consists of tables and figures, without which it is difficult to draw a discussion and conclusion from the article. [file 2299725.f1.docx]

Dear Editor the Scientific World Journal ,

The supplemental file that I could think of as follows:

**Method**

**Tools:** The tools used in this research were magnetic stirrer (Yellow line), pH meter (pH 108 ATC), Brookfield viscometer (DV-E), homogenizer (WiseTis), oven (Memmert), analytical balance (Mettler Toledo), other tools commonly used in Pharmacognosy & Natural Materials Laboratory, and in Pharmacy & Formulation Technology Laboratory

**Material:** The materials used in this study were the water fraction of cocoa pods, 70% ethanol, 96% ethanol, stearic acid, triethanolamine, glycerin, liquid paraffin, nipagin, nipasol, aquades, N-hexane, ethyl acetate, sodium metabisulfite. All chemicals were of analytical grade.

**Test Animal (Rabbit):** Experimental animals are animals that are bred to be used as model animals in research to study and develop various fields of science. The use of experimental animals aims to examine reactions, interactions as well as the impact of the material being tested on animals thoroughly and in depth. In this study, rabbits were used as test animals for the preparation of creams to stimulate hair growth. The Rabbits used in the study was : *Oryctolagus cuniculus.*

Treatment of Cream Preparations on rabbit’s back: The back of the rabbit is divided into several plots as follows:

a. Box 1: normal control (no treatment)

b. Box 2: positive control (minoxidil 5.0%)

c. Box 3: negative control control (cream base)

d. Box 4: cream preparation of cocoa pod peels water fraction (7.5%)

e. Box 5: cream preparation of cocoa pod peels water fraction (10.0%)

f. Box 6: preparation of cocoa pod peels water fraction cream (12.5%)

Determination of the smearing area on the rabbit's back was carried out randomly, and it was hoped that hair growth activity in all regions with different representatives could be represented

**Example of the progress of rabbit hair growth activity during the experiment (rabbit 1)**

|  | 3^rd^ day | 6^th^ day | 9^th^ day | 12^th^ day | 15^th^ day | 18^th^ day |
| --- | --- | --- | --- | --- | --- | --- |
| Control (normal) |  |  |  |  |  |  |
| Control (negative) |  |  |  |  |  |  |
| Control (positive) |  |  |  |  |  |  |
| Cream (7.5%) |  |  |  |  |  |  |
| Cream (10.0%) |  |  |  |  |  |  |
| Cream (12.5%) |  |  |  |  |  |  |

**Example of STATISTICAL DATA PROCESSING RESULTS:**

A. Statistical Test Results Cream preparations

1. Normality Test

Objective: To determine the normality distribution of the average hair length of rabbits in testing the water fraction of the nail munding root

Hypothesis

Ho : the average hair length of rabbits is normally distributed

H1 : the average hair length of rabbits is not normally distributed: 0.05

Test stats : Shapiro Wilk

Criteria: Ho is rejected if the significance value is <0.05

Ho is accepted if the significance value is > 0.05

|  | Component | Kolmogorov-Smirnov^a^ | | | Shapiro-Wilk | | |
| --- | --- | --- | --- | --- | --- | --- | --- |
|  |  | Statistic | df | Sig. | Statistic | df | Sig. |
| Hair length | Control (normal) | .283 | 3 | . | .935 | 3 | .506 |
|  | Control (negative0 | .364 | 3 | . | .800 | 3 | .114 |
|  | Control (positive) | .337 | 3 | . | .854 | 3 | .250 |
|  | Cream( 7.5%) | .359 | 3 | . | .811 | 3 | .142 |
|  | Cream (10.0%) | .295 | 3 | . | .920 | 3 | .451 |
|  | Cream (12.5%) | .251 | 3 | . | .966 | 3 | .646 |

Conclusion: Ho is accepted, so the data on the average hair length of rabbits from each group is normally distributed

2. Homogeneity Test

Objective : To determine the homogeneity distribution of the average hair length of rabbits on the cream of cocoa pod peel water fraction testing

Hypothesis

Ho : the average hair length of rabbits is normally distributed

H1 : the average hair length of rabbits is not normally distributed

: 0.05

Test statistics : Levene's test

Criteria: Ho is rejected if the significance value is <0.05

Ho is accepted if the significance value is > 0.05

| Hair length | | | |
| --- | --- | --- | --- |
| Levene Statistic | df1 | df2 | Sig. |
| .544 | 7 | 16 | .789 |

3. ANOVA test

Objective: to find out there is a significant difference in the average hair length of rabbits in each group

Hypothesis:

Ho : There is no significant difference in the average hair length of rabbits in each group

H1 : There is a significant difference in the average hair length of rabbits in each group

α	: 0,05

Test statistics: ANOVA

Criteria:

Ho is rejected if the significance value is < 0.05

Ho is accepted if the significance value is > 0.05

Results :

| **ANOVA** | | | | | |
| --- | --- | --- | --- | --- | --- |
| Hair length | | | | | |
|  | Sum of Squares | Df | Mean Square | F | Sig. |
| Between Groups | .175 | 7 | .025 | .117 | .996 |
| Within Groups | 3.414 | 16 | .213 |  |  |
| Total | 3.590 | 23 |  |  |  |

Conclusion: H0 is accepted, which means that there is no significant difference in the average hair length of rabbits in each group

Thanks and rgds,

Prof. Resmi Mustarichie
